# Association between playing cards/mahjong and risk of incident dementia among the Chinese older adults: a prospective cohort study

**DOI:** 10.3389/fnagi.2022.966647

**Published:** 2022-08-22

**Authors:** Gang Tian, Jingliang Shuai, Rui Li, Tong Zhou, Yan Shi, Gang Cheng, Yan Yan

**Affiliations:** Department of Epidemiology and Medical Statistics, Xiangya School of Public Health, Central South University, Changsha, China

**Keywords:** dementia, cognitively stimulating activities, playing cards/mahjong, Chinese older adults, prospective study

## Abstract

**Objectives:** Studies have shown that the frequent participation of the elderly in cognitive stimulation activities is associated with a reduced risk of dementia, but the prospective evidence of this association is limited.

**Methods:** We used data from a prospective cohort study of the Chinese Longitudinal Healthy Longevity Survey (CLHLS), and included 11,821 community-living Chinese individuals aged 65 years or older at 2008 baseline who were free of dementia, and were followed up every 2–3 years until 2018. Cox proportional hazards models were applied to generate the hazard ratios (HRs) and 95% confidence intervals (CIs) for analyzing the associations between the frequency of playing cards/mahjong and the incidence of dementia.

**Results:** A total of 821 participants were diagnosed with dementia during the 10-year follow-up. The average age of patients with dementia and non dementia were 89 and 90 years old, respectively. Compared with participants who rarely or never played cards/mahjong, participants who played cards/mahjong almost every day had a significantly lower risk of dementia (HR = 0.63; 95%CI, 0.42–0.95) after the multivariable-adjusted model. Similar results were observed in subgroup analyses based on sex (male: HR = 0.52, 0.28–0.96; female: HR = 0.62, 0.36–0.98), age (<85years: HR = 0.55, 0.32–0.89), regularly exercise (yes: HR = 0.44, 0.28–0.87) and MMSE score [above median (25): HR = 0.66, 0.41–0.92].

**Conclusions:** Playing cards/mahjong in the elderly may contribute to reducing the risk of dementia.

## Introduction

Dementia, as a central nervous degenerative disease in middle-aged and older adults, can cause progressive cognitive impairment and behavioral damage, and often causes great pain to the elderly over 65 years old all over the world (Li et al., 2015; Charlson et al., 2016; GBD 2016 Dementia Collaborators, 2019). With the considerable rise in life expectancy and the aggravation of the aging process, the number of individuals with dementia is increasing. An estimated population of more than 46 million people worldwide suffered from dementia in 2016. The number is expected to exceed 130 million by 2050 (GBD 2016 Dementia Collaborators, 2019), and Chinese dementia patients account for about 25% of the total number of dementia patients in the world (Alzheimer’s Disease International, 2015). Because dementia cannot be cured, it will bring a huge burden to the country, healthcare professionals, and family members. Therefore, it is necessary to explore some effective methods to prevent dementia in the elderly and delay the onset of the disease.

As we all know, mahjong and cards are very popular in China, and they are also one of the favorite leisure and entertainment activities for the elderly. Previous studies conducted in North America and Europe (Wilson et al., 2002a, b; Akbaraly et al., 2009; Paillard-Borg et al., 2009; Yates et al., 2016) showed that the active participation of the elderly in leisure and recreational activities, especially intellectual activities, such as reading, playing chess or playing cards, can improve cognitive reserve, protect and restore cognitive function, and help reduce the risk of dementia in the elderly (Fratiglioni et al., 2004; Valenzuela et al., 2007; Cheng, 2016; Nelson et al., 2021). However, researches on this aspect are still scarce in China (Lee et al., 2018), and relevant issues are worth exploring. Those who often actively participate in intellectual activities usually pay more attention to health and have a healthier lifestyle, such as regular exercise, a balanced diet and less smoking. These factors have been shown to be beneficial in preventing dementia (Lovden et al., 2013; Di Marco et al., 2014; Hussenoeder and Riedel-Heller, 2018; Lee et al., 2019). Whether participation in intellectual activities, particularly mahjong/cards in China, can prevent and delay the onset of dementia independently of these adverse lifestyles remains to be determined. Hence, We used the 10-year follow-up data of the Chinese Longitudinal Healthy Longevity Survey (CLHLS) for individuals aged 65 and above (Gu et al., 2017) to investigate the associations between the frequency of playing cards/mahjong and dementia. The long interval between the assessment of exposure factors and the diagnosis of dementia may make us more confident about the time effect and causality of this association (Scarmeas and Stern, 2003).

Our study objectives were to estimate the hazard ratios (HRs) for dementia from the different frequencies of playing cards/mahjong, using never playing cards/mahjong as a reference; estimated dementia risk across age, sex, regular exercise status, and the Mini-Mental State Examination (MMSE) score stratifications; to ensure the stability and reliability of our results, we conducted sensitivity analysis by excluding participants who were MMSE scores <18 at baseline, further excluding participants suffering from epilepsy or stroke at baseline, and participants suffering from dementia during the first year of follow-up in turn.

## Methods

### Study design and participants

The CLHLS study is an ongoing, population-based, prospective cohort study that was conducted in 1998 and randomly selected half of the counties or cities in 22 of 31 provinces in China. These areas include approximately 85% of the total Chinese population (National Bureau of Statistics, 2012). Our study analysis selects data from 2008 to 2018 waves and mainly collects information on the health status and quality of life of older adults individuals aged 65 and older. Details of the study design and methods have been described elsewhere (Deng et al., 2020).

**Figure 1:**
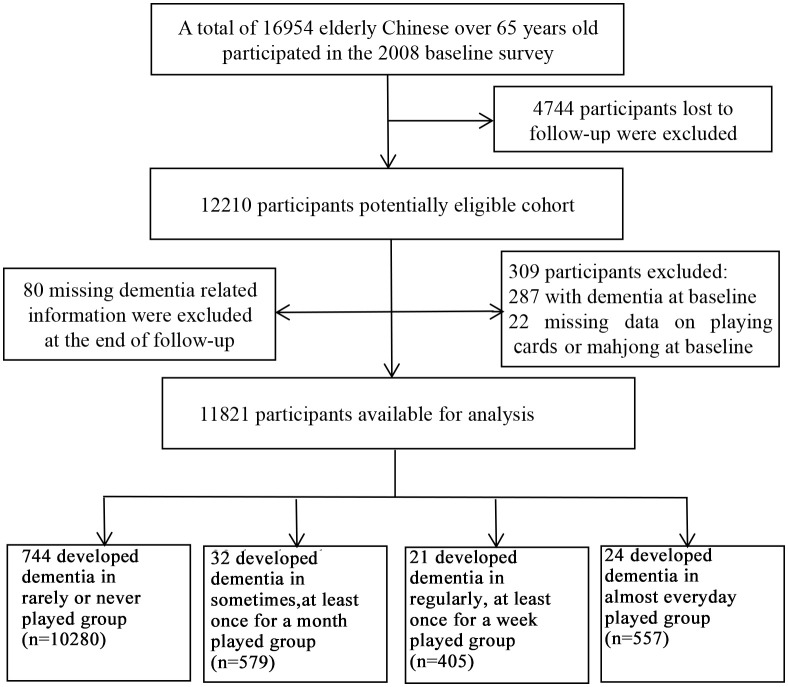
Flow chart of the study population.

The CLHLS data were collected by trained staff through face-to-face interviews with the older adults themselves or their relatives or caregivers. After evaluation, the data quality and the report information reliability of the CLHLS are reasonably good (Zeng et al., 2008). A total of 16,954 Chinese elderly over 65 years old participated in the 2008 baseline survey, which included rich information about playing cards/mahjong and dementia. After excluding cases with missing information on key variables, with dementia at baseline and loss to follow-up, the final 11,821 participants available were included for the analysis of the association of frequency of playing cards/mahjong and dementia. The full screening process of research participants is presented in Figure 1.

### Assessment of frequency of playing cards/mahjong

To obtain the data on playing mahjong/cards status at the baseline, we invited participants themselves or a close relative of the interviewee to answer some questions, such as “do you now perform the following activities regularly?”, with options of “almost every day”, “not every day, but at least once a week”, “not every week, but at least once a month”, “not every month, but sometimes”, and “rarely or never”. Based on the frequency of playing mahjong/cards in the 2008 baseline survey, Finally, we classified four types of mahjong/card players. Those who “not every month, but sometimes” or “never” played mahjong/cards at the baseline were classified as “rarely or never players”, which was the reference category in the regression analyses.

### Identification of dementia

The outcome of this study was incident dementia in the following up to 10 years. The identification of individuals with dementia came from reports through family members or caregivers, diagnosed by qualified medical institutions, or taking dementia treatment drugs. For those participants who were dead during follow-up, we asked their family members or caregivers whether they had dementia before death.

### Evaluation of other variables

To control the potential confoundings, covariates, such as socio-demographic information, lifestyle factors, and health status, were included in our analyses. Socio-demographic variables included age (continuous), sex, education (no schooling received/1 year or more), household income [low (<10,000 RMB); middle (10,000–30,000 RMB); high (>30,000 RMB)], marital status (married/unmarried), living with household members (yes/no). Lifestyle factors included smoking status (current smoker/current non-smoker), drinking status (current drinker/current non-drinker), and regular exercise (yes/no). Health status information included body mass index (BMI) [underweight (<18.5), normal (18.5–23.9), overweight (24–27.9), obese (≥28)] (Lv et al., 2018). The identification of individuals with hypertension or diabetes came from reports through family members or caregivers, diagnosed by qualified medical institutions, or taking hypertension or diabetes treatment drugs. Because of the close relationship between cognitive function and dementia (Petersson and Philippou, 2016), we carefully analyzed the role of cognitive function in dementia. Cognitive function was measured by the Chinese version of the Mini-Mental State Examination (MMSE) in the CLHLS. The validity and reliability of the Chinese MMSE have been verified (Zeng et al., 2017). The MMSE score ranged from 0 to 30, and a higher score indicated better cognitive function (Yi and Vaupel, 2002; Lv et al., 2019). A score below 18 was considered to be a cognitive impairment among older adults in China (Hao et al., 2018; Mao et al., 2020).

### Statistical analysis

Baseline characteristics were described as medians and interquartile ranges (IQR) for continuous variables (not normally distributed), the count and percentage for categorical variables according to different groups of playing mahjong/cards frequency and with and without incident dementia. The χ^2^ test was used for comparing differences among groups in categorical variables and the Kruskal-Wallis H test or the Mann-Whitney test in continuous variables. We calculated crude incidence rates (IR; per 1,000 person-years) of dementia across categories of playing cards/mahjong frequency. The person-years were calculated by summing each participant’s contribution to follow-up time (from baseline to the year of assessment when the participant was found to have dementia, or to the year of the last assessment if the participant remained free of dementia). All analyses were performed using R version 4.0.5 (R Foundation for Statistical Computing), and all reported *P* < 0.05 (2-tailed) were considered statistically significant.

Cox proportional hazards models were applied to generate the hazard ratios (HRs) and 95% confidence intervals (CIs) for analyzing the associations between the frequency of playing cards/mahjong and the risk of dementia. Initially, we constructed models without any adjusted covariates (unadjusted model), additionally adjusted only for age and sex (age and sex-adjusted model), and further adjusted for education, household income, marital status, smoking status, drinking status, regular exercise, BMI, living with family members, history of hypertension and diabetes and MMSE score (multivariable-adjusted model).

To further explore the influence of sex, age, regular exercise, and MMSE score (median) on the association of the frequency of playing cards/mahjong with the risk of dementia, we conducted subgroup analyses. To ensure the stability and reliability of our study results and exclude more interference, we carried out sensitivity analysis, first excluding participants who were MMSE scores <18 at baseline, then further excluding participants who suffer from epilepsy or stroke at baseline, and finally excluding dementia that occurred during the first year of follow-up.

## Results

### Participant characteristics

A total of 11,821 participants were included in our study (Figure 1). As shown in Table 1, at baseline, compared with other groups, those participants who rarely or never played cards/mahjong were older, with a median (IQR) age of 90 (81–98) years, more female (60.1%), lower married rate (26.3%), and lower MMSE score (24 points). With the increasing frequency of playing cards/mahjong (divided into four groups), the proportion of educated (34.0%, 42.9%, 46.2%, and 49.2%, respectively) smokers (34.0%, 42.9%, 46.2%, and 49.2%, respectively), drinkers (34.0%, 42.9%, 46.2%, and 49.2%, respectively) and regular exercisers (34.0%, 42.9%, 46.2%, and 49.2%, respectively) also increased among participants (shown in Table 1). There was no significant difference in the proportion of living with family members and suffering from hypertension and diabetes among the groups. A total of 821 participants developed incident dementia during the 10-year follow-up. As summarized in Table 1, those who developed incident dementia were older [age: median (IQR), 90 (82–96)] than those who remained free of dementia [age: median (IQR), 89 (79–97)], and were predominantly female [530 (64.6%) vs. 6,248 (56.8%)]; with a significantly higher percentage of uneducated [629 (76.6%) vs. 8,013 (72.9%)], married, smokers, drinkers, and less MMSE score, with a higher prevalence of hypertension, diabetes.

**Table 1:** Characteristics of the study participants according to the frequency of playing cards/mahjong and Dementia status at end of follow-up at baseline.

**Characteristics**	**Frequency of playing cards/mahjong**					**Dementia status at end of follow-up**		
	**Rarely or never**	**Sometimes, at least once a month**	**Regularly, at least once a week**	**Almost everyday**	***P* value for difference**	**No dementia**	**Dementia**	***P* value for difference**
Participant (n)	10,280	579	405	557	11,000	821		
Age (year), median (IQR)	90 (81–98)	81 (71–89)	88 (80–94)	80 (71–88)	<0.001	89 (79–97)	90 (82–96)	0.0052
Sex, Female (n, %)	6,175 (60.1)	217 (37.5)	158 (39.0)	228 (41.0)	<0.001	6,248 (56.8)	530 (64.6)	<0.001
No schooling received (n, %)	7,810 (76.0)	331 (57.1)	218 (53.8)	283 (50.8)	<0.001	8,013 (72.9)	629 (76.6)	0.0188
Married (n, %)	2,706 (26.3)	280 (48.3)	187 (46.1)	267 (48.0)	<0.001	3,249 (29.5)	191 (23.3)	<0.001
Household income (RMB)					<0.001			0.4584
Low (≤10,000)	4,666 (45.4)	190 (32.8)	142 (35.1)	214 (38.4)		4,836 (44.0)	376 (45.8)	
Middle (10,000–30,000)	3,851 (37.5)	256 (44.2)	183 (45.2)	253 (45.4)		4,244 (38.6)	299 (36.4)	
High (≥30,000)	1,763 (17.2)	133 (23.0)	80 (19.7)	90 (16.1)		1,920 (17.5)	146 (17.8)	
Current smoker (n, %)	1,619 (15.8)	144 (24.9)	136 (33.6)	188 (33.8)	<0.001	1,967 (17.9)	120 (14.6)	0.0179
Current drinker (n, %)	1,679 (16.3)	152 (26.2)	113 (28.0)	178 (32.0)	<0.001	1,996 (18.2)	126 (15.4)	0.0439
Regular exercise (n, %)	2,322 (22.5)	215 (37.1)	171 (42.2)	278 (59.9)	<0.001	2,782 (25.3)	193 (23.5)	0.2561
Living with family members, (n, %)	8,596 (83.6)	488 (84.3)	336 (83.0)	452 (81.2)	0.4432	9,204 (83.7)	668 (81.4)	0.0855
BMI (kg/m^2^) group (n, %)					<0.001			0.8329
Underweight (<18.5)	3,892 (37.9)	128 (22.1)	90 (22.2)	135 (24.2)		3,959 (36.0)	286 (34.9)	
Normal (18.5–23.9)	5,164 (50.2)	351 (60.6)	242 (59.8)	322 (57.8)		5,653 (51.4)	426 (51.9)	
Overweight (24–27.9)	953 (9.3)	84 (14.5)	58 (14.3)	84 (15.1)		1,091 (9.9)	88 (10.7)	
Obese (≥28)	271 (2.6)	16 (2.8)	15 (3.7)	16 (2.9)		297 (2.7)	21 (2.6)	
MMSE score, median (IQR)	24 (13–29)	29 (26–30)	29 (26–30)	27 (24–29)	<0.001	25 (16–29)	23 (11–28)	<0.001
MMES<18 (n, %)	3,255 (31.7)	34 (5.9)	25 (6.2)	19 (3.4)	<0.001	3,037 (27.6)	296 (36.1)	<0.001
MMES >= 18 (n, %)	7,025 (68.3)	545 (94.1)	380 (93.8)	538 (96.6)		7,963 (72.4)	525 (63.9)	
Hypertension (n, %)	1,873 (18.2)	108 (18.7)	89 (22.0)	119 (21.3)	0.0781	2,010 (18.3)	179 (21.8)	0.0120
Type 2 diabetes (n, %)	192 (1.9)	15 (2.6)	9 (2.2)	19 (3.4)	0.0516	216 (2.0)	19 (2.3)	0.4875

Data are n, median (IQR), or n (%).

### Association of playing cards/mahjong with risk of dementia

Overall, with the increase in playing cards/mahjong frequency, the crude rate of dementia events decreased gradually. The incidence rate (95%CI) per 1,000 person-year in the rarely or never group, sometimes, at least once for a month group, regularly, at least once for a weekly group, and the almost everyday group were 16.4 (15.3–17.6), 8.8 (6.2–12.4), 8.2 (5.4–12.5) and 6.7 (4.5–9.9), respectively (Table 2).

**Table 2:** Associations between frequency of playing card/mahjong and dementia for Chinese older adults.

**Frequency of playing card/mahjong**	**N cases/N total**	**ID (95%CI) per 1,000 persons-years**	**Unadjusted model HR(95% CI)**	***P* value**	**Age-and sex-adjusted model HR(95% CI)**	***P* value**	**Multivariable-adjusted model HR (95% CI)**	***P* value**
Rarely or never	744/10,280	16.4 (15.3–17.6)		Ref		Ref		Ref	
Sometimes, at least once for a month	32/579	8.8 (6.2–12.4)	0.52 (0.36–0.73)	0.002	0.77 (0.54–1.10)	0.139	0.84 (0.59–1.21)	0.146
Regularly, at least	21/405	8.2 (5.4–12.5)	0.47 (0.31–0.74)	<0.001	0.66 (0.42–1.02)	0.087	0.75 (0.50–1.16)	0.134
once for a week								
almost everyday	24/557	6.7 (4.5–9.9)	0.38 (0.25–0.57)	<0.001	0.55 (0.36–0.82)	0.016	0.63 (0.42–0.95)	0.031

ID, Incidence density; HR, hazard ratio; CI, Confidence interval. Multivariable-adjusted model including age, sex, education, household income, marital status, smoking status, drinking status, exercise, BMI, living with family members, hypertension, diabetes, and MMSE score.

In Cox proportional hazards models reported in Table 2; the corresponding unadjusted HR (95%CI) for dementia was significantly lower in those who almost every day played cards/mahjong (HR = 0.38; 95%CI, 0.25–0.57) than those who rarely or never played cards/mahjong. It was associated with the risk of dementia after adjusting for sex and age (HR = 0.55; 95%CI, 0.36–0.82). It remained significant after further adjusting for additional education, household income, marital status, smoking status, drinking status, regular exercise, BMI, living with family members, hypertension, diabetes, and MMSE score (HR = 0.63; 95%CI, 0.42–0.95). There was no clear evidence of an association between the other two groups.

### Subgroup analysis

**Figure 2:**
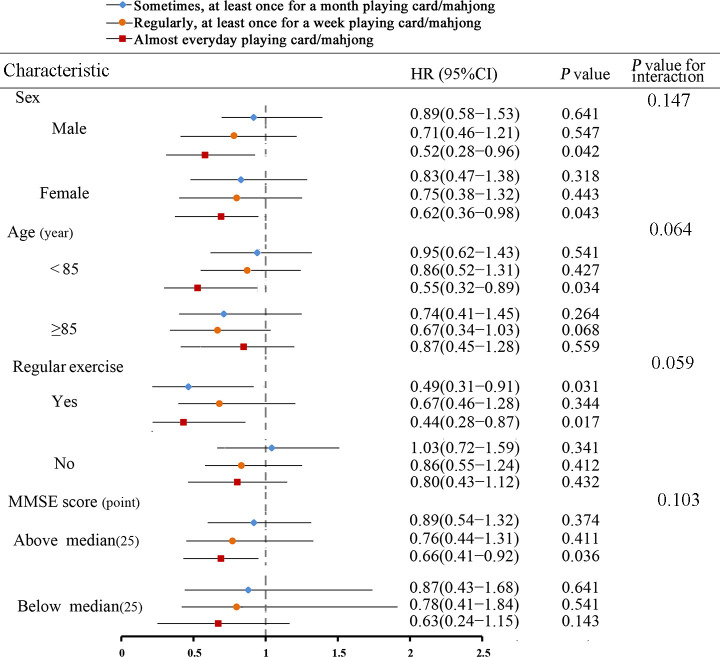
Hazard ratios (HRs) and confidence intervals (CIs) for the association between frequency of playing cards/mahjong and dementia, with rarely or never playing cards/mahjong as a reference group, by baseline sex, age, regular exercise, and MMSE score, adjusting for baseline age, sex, education, household income, marital status, smoking status, drinking status, exercise, BMI, living with family members, hypertension, diabetes, and MMSE score.

We performed subgroup analysis by sex, age, regular exercise, and MMSE score median. In the sex-stratified analysis, the risk of dementia in almost everyday playing cards/mahjong group (male: HR = 0.52, 0.28–0.96; female: HR = 0.62, 0.36–0.98) was lower than that in other the frequency of playing cards/mahjong groups. In age-stratified analysis, the risk of dementia in almost everyday playing cards/mahjong group in less than 85 years (HR = 0.55, 0.32–0.89) was lower than in more than 85 years (HR = 0.87, 0.45–1.28). Similar pattern was observed in regular exercise-stratified analysis (yes: HR = 0.44, 0.28–0.87), and MMSE score-stratified analysis [above median (25): HR = 0.66, 0.41–0.92] (Figure 2). There is no evidence that there is an interaction between whether the elderly play mahjong/cards and sex, age, regular exercise, and MMSE score median groups.

### Sensitivity analysis

The results of the sensitivity analyses are presented in Table 3. We still used three models (unadjusted, age- and sex-adjusted, multivariable-adjusted) to analyze the association of the frequency of playing cards/mahjong with the risk of dementia. The unadjusted, age- and sex-adjusted, and multivariable-adjusted models excluded participants who were MMSE scores <18 at baseline, further excluding participants who suffer from epilepsy or stroke at baseline, or additionally excluding dementia that occurred during the first year of follow-up did not make a big difference to the original results.

**Table 3:** Association of frequency of playing cards/mahjong with dementia after further exclusions.

**Frequency of playing cards/mahjong**	**Incidence density per 1,000 persons-years**	**Unadjusted model HR (95% CI)**	***P* value**	**Age-and sex-adjusted model HR (95% CI)**	***P* value**	Multivariable-adjusted model* HR (95% CI)	***P* value**
Excluding participants who were MMSE scores < 18 at baseline							
Rarely or never	12.4 (11.3–13.6)	Ref		Ref		Ref	
Sometimes ^a^	8.4 (5.7–12.2)	0.60 (0.42–0.87)	0.008	0.83 (0.57–1.20)	0.132	0.72 (0.41–1.27)	0.146
Regularly ^b^	7.7 (4.8–12.0)	0.58 (0.38–0.90)	0.013	0.75 (0.49–1.16)	0.138	0.61 (0.34–1.11)	0.094
almost everyday	6.6 (4.3–10.1)	0.47 (0.31–0.71)	<0.001	0.62 (0.41–0.94)	0.011	0.54 (0.36–0.97)	0.021
Further excluding participants who suffer from epilepsy or stroke at baseline							
Rarely or never	12.0 (10.8–13.1)	Ref		Ref		Ref	
Sometimes ^a^	7.8 (5.3–11.4)	0.57 (0.39–0.84)	0.006	0.80 (0.54–1.19)	0.132	0.73 (0.40–1.32)	0.326
Regularly ^b^	7.6 (4.8–11.9)	0.59 (0.38–0.92)	0.014	0.77 (0.49–1.21)	0.137	0.69 (0.38–1.26)	0.294
almost everyday	6.7 (4.4–10.1)	0.48 (0.32–0.74)	<0.001	0.65 (0.43–0.98)	0.031	0.60 (0.29–1.22)	0.308
Excluding dementia that occurred during the first year of follow-up							
Rarely or never	12.0 (11.0–13.1)	Ref		Ref		Ref	
Sometimes ^a^	7.9 (5.5–11.4)	0.74 (0.50–1.10)	0.105	1.05 (0.70–1.56)	0.096	0.90 (0.48–1.68)	0.279
Regularly ^b^	7.1 (4.5–11.2)	0.68 (0.42–1.10)	0.107	0.89 (0.55–1.45)	0.073	0.69 (0.32–1.50)	0.236
almost everyday	5.1 (3.2–8.0)	0.48 (0.30–0.79)	0.004	0.65 (0.40–1.05)	0.066	0.63 (0.31–1.31)	0.195

HR, hazard ratio; CI, Confidence interval. Sometimes ^a^: at least once for a month; Regularly ^b^: at least once for a week. Multivariable-adjusted model^*^ including age, sex, education, household income, marital status, smoking status, drinking status, exercise, BMI, living with family members, hypertension, diabetes, and MMSE score.

## Discussion

This extensive community-based, prospective cohort study showed that more frequency of playing cards/mahjong was associated with a significant reduction in the risk of dementia among older adults individuals aged 65 and older. Furthermore, we found that the protective association was stronger for older adults who reported playing cards/mahjong almost every day with regular exercise than those without regular exercise. We did not find any significant difference in age, sex, and MMSE score median, and further sensitivity analysis yielded no substantial changes in our findings after adjusting confounding factors.

The present findings align with several previous studies that active participation in stimulating intellectual activities (such as playing cards or checkers), especially among older adults, is associated with better cognitive functioning (Leung et al., 2010). In addition, some studies had also found that more frequently playing stimulating intellectual games (such as cards, bingo, chess, mahjong, and crossword puzzle) were associated with a decreased risk of dementia (Wang et al., 2006; Qiu et al., 2019; Sommerlad et al., 2020). The reasons why these stimulating intellectual games can reduce the risk of dementia may be explained by the fact that playing cards or mahjong (game activities welcomed by the Chinese circle) is a strong and comprehensive stimulating activity in cognitive domains (involving attention, reasoning, memory, and initiative capacity; Marioni et al., 2015). Some studies have found that social isolation and loneliness are also risk factors for dementia (Shankar et al., 2013; Rafnsson et al., 2020; Shen et al., 2022). Meanwhile, playing cards or mahjong is a group entertainment and can not only promote communication between the elderly but also give them good social support and emotional comfort, which is also proven to help to reduce the risk of dementia or cognitive impairment (Wang et al., 2012; Raz et al., 2016). However, as we know, many previous studies did not fully consider other confounding factors (such as education level and whether live with family members) and baseline cognitive status, both of which can lead to potentially biased results. Our finding was consistent with a recent prospective study result that active participation in playing cards/mahjong might be in favor of decreasing the risk of dementia in older adults (Lee et al., 2018). However, the study was followed-up for only 3 years, and relevant evidence showed that dementia was considered to develop slowly over many years (Laurin et al., 2001), so the causality of this study’s conclusion was still controversial. Additionally, most studies had incorporated various types of stimulating intellectual activities as independent variables, while our study included playing cards/mahjong as the only factor to explore the independent impact on the risk of dementia in older adults through adjusting as many confounding factors as possible.

Some prospective studies also investigated the relationship between other types of leisure activities (such as social, entertainment and physical) and cognitive decline or dementia, but they did not determine the frequency of social or entertainment participation, or physical activities (Wilson et al., 2002b; Lee et al., 2018; Kim et al., 2021). These results were contrary to the relationship between stimulating intellectual activities and cognitive decline or dementia, but these findings were significant because they showed that the association of cognitive decline or dementia risk reflected mental stimulation rather than nonspecific results of other types of leisure activities. Compared with stimulating intellectual activities, these activities are usually more passive and have less cognitive involvement, so we deduce that stimulating intellectual activities might be more effective than engaging in various nonintellectual leisure activities (such as recreational, social, and physical) in preventing dementia.

There is no consensus on the potential mechanism of stimulating intellectual activities and preventing dementia. One hypothesis is that, frequent participation in cognitively stimulating activities can protect the cognitive function of older adults from decline (Wilson et al., 2000) because repeating some cognitive skills can make neurons more active and less vulnerable to disruption by dementia pathology (Alexander et al., 1997). Another similar view points out that frequent cognitive stimulating activity may strengthen thought processing skills, such as ratiocination, calculation, and perceptual speed, which may be conducive to compensating and resist for age-related decline in other cognitive systems (Beyer et al., 2021). Some studies had explored possible biological mechanisms for the association of cognitively stimulating activities with cognitive function (Stern, 2012). These findings manifested that being mentally active may delay the onset of clinical dementia by improving cognitive reserve. According to cognitive reserve theory in neuroscience, people with a higher level of cognitive reserve can buffer the effects of neuropathology in brain anatomical substrate and function, and also have greater dynamic neural network compensation such that the larger cognitive reserve, the more serious the pathological damage needed to cause functional impairment (Richards and Deary, 2005). A recent study showed that cognitive stimulation can improve functional connectivity between the hippocampus and superior frontal cortex to resist cognitive function decline (Suo et al., 2016). The above findings show that cognitive training may improve cognition, possibly through different neural regulation mechanisms, which can explain our findings that more frequency of playing mahjong/cards lowers the risk of dementia.

Regarding the strengths of this study, it is a community-based, national-wide prospective design, a large sample of over 65 older adults, a relatively long follow-up period with about 3 years of evenly spaced observations per individual, and adjustment for established and potential confounders. Of course, our study also has some limitations. First, our findings only apply to older adults in China. Due to the cognitive function effects of playing cards/mahjong may be different between young and older adults individuals, this effect should be carefully extended to young people. Second, information on covariates and dementia status is collected through self-reported in the form of a questionnaire, thus, recall bias and information bias are possible, and some undiagnosed dementia cases may be omitted, which may affect the research results. Third, As the subjects of our study were older and the number of deaths was as high as 9,318 in the 10-year follow-up period, although we repeatedly confirmed to the family members whether the deceased had been diagnosed with dementia, some family members still did not give a clear response, which made us miss some dementia cases and had a certain impact on the outcome. Fourth we do not control other types of leisure activities. Although this is also important, it does not alter the main results in that frequent playing cards/mahjong is associated with a significantly decreased risk of dementia.

## Conclusion

Our study provides evidence that frequently playing cards/mahjong may decrease the risk of dementia among Chinese elderly over 65 years old. Given China’s huge population base and increasing aging population, proper and rational participation of the elderly in this activity could provide a certain degree of social support and emotional comfort.

## Data Availability Statement

The raw data supporting the conclusions of this article will be made available by the authors, without undue reservation.

## Ethics Statement

The datasets supporting this article are publicly available from the project of the CLHLS. The study was approved by the Research Ethics Committee of Peking University (IRB00001052-13074). All study participants or their legal proxy respondents must obtain and sign written informed consent before completing each study questionnaire.

## Author Contributions

GT participated in the design, data analysis, and writing of this study. YY provided supervision and guidance at all stages including the analyses. JS, RL, and TZ assisted with drafting the article. YS and GC prepared the manuscript for publication. All authors reviewed and commented on drafts of the manuscript. All authors contributed to the article and approved the submitted version.

## Funding

The Chinese Longitudinal Healthy Longevity Survey (CLHLS) has been supported by NIA/NIH grants R01 AG023627-01 and P01 AG008761, awarded to Duke University, with Chinese matching support for personnel costs and some local expenses. The CLHLS was supported by funds from the US National Institute on Aging (NIA), the China Natural Science Foundation, the China Social Science Foundation, and the United Nations Fund for Population Activities (UNFPA) and was managed by the Center for Healthy Aging and Development Studies, Peking University.
